# Downregulation of ATP1A1 promotes cancer development in renal cell carcinoma

**DOI:** 10.1186/s12014-017-9150-4

**Published:** 2017-05-04

**Authors:** Dan Zhang, Peng Zhang, Pengbo Yang, Yu He, Xixi Wang, Yanfang Yang, Hongxia Zhu, Ningzhi Xu, Shufang Liang

**Affiliations:** 1 State Key Laboratory of Biotherapy and Cancer Center, West China Hospital, Sichuan University, and Collaborative Innovation Center for Biotherapy, No.17 Section 3, People’s South Road, Chengdu, 610041 People’s Republic of China; 2 Department of Urinary Surgery, West China Hospital, West China Medical School, Sichuan University, Chengdu, 610041 People’s Republic of China; 3 Laboratory of Cell and Molecular Biology and State Key Laboratory of Molecular Oncology, Cancer Institute and Cancer Hospital, Chinese Academy of Medical Sciences, Beijing, 100021 People’s Republic of China

**Keywords:** Na^+^/K^+^-ATPase α1 subunit, Downregulation, Renal cell carcinoma

## Abstract

**Background:**

Aberrant expression of Na^+^/K^+^-ATPase α1 subunit (ATP1A1) is widely observed in multiple types of tumors, and its tissue-specific expression relates to cancer development. However, the functions and molecular mechanisms in renal cell carcinoma (RCC) are not fully understood.

**Methods:**

We investigated the ATP1A1 expression changes and possible roles in RCC through a quantitative proteomic approach and an integrative biochemical assessment. We detected ATP1A1 in RCC with LC–MS/MS, and further validated its expression with immunohistochemical analyses of 80 pairs of the RCC tumor and non-tumor tissues samples. The association of ATP1A1 expression with RCC pathology was statistically analyzed. Cell proliferation, migration and apoptosis were measured by CCK-8, boyden chamber assay and flow cytometry, respectively. The production of reactive oxygen species (ROS) was labeled with a single staining using a commercial kit, and was further detected with flow cytometry.

**Results:**

The ATP1A1 shows a significantly decreased expression in human RCC tissues than in the adjacent non-tumor tissues. The RCC patients with ATP1A1-positive expression exhibit longer overall survival time than the ATP1A1-negative patients. The exogenous overexpression of ATP1A1 inhibits RCC cell proliferation and cell migration by increasing the production of ROS. In addition, ATP1A1-mediated Raf/MEK/ERK signaling pathway is suppressed in RCC cells, indicating the possible occurrence of induced cell apoptosis.

**Conclusions:**

Our in vitro and in vivo data of ATP1A1 inhibitory roles in RCC progression suggest that ATP1A1 is a potential novel suppressor protein for renal cancer.

**Electronic supplementary material:**

The online version of this article (doi:10.1186/s12014-017-9150-4) contains supplementary material, which is available to authorized users.

## Background

Renal cell carcinoma (RCC) is one of the most common type of kidney cancer. It originates from the proximal tubular epithelium, and is a lethal genitourinary cancer, which accounts for about 3% of all adult malignancies, including 209,000 new cases and 102,000 deaths worldwide every year [1, 2]. Regarding RCC therapy, tumor metastasis is a frequent clinic issue, and the treatment for metastatic and locally unresectable RCC remains challenging [3]. Investigation of protein-mediated molecular mechanism is helpful for exploring RCC carcinogenesis and metastasis.

The Na^+^/K^+^-ATPase is an electrogenic ion pump, which transports three sodium ions out and two potassium ions into cell per pump cycle at expense of one ATP molecule, thereby generating a transmembrane sodium gradient across the plasma membrane [4, 5]. The sodium gradient is crucial for efficient functioning of other Na^+^ coupled transport systems and provides primary energy for uptake and extrusion of a wide variety of solutes by epithelial cells. Na^+^/K^+^-ATPase is a membrane bound protein. It is composed of four α- and three β-subunit isoforms identified in mammalian cells [6–8]. Among these isoforms, the Na^+^/K^+^-ATPase α_1_ subunit (ATP1A1) is a catalytic subunit which bears Mg2^+^, ATP, Na^+^, K^+^ and ouabain binding sites [8]. The β1 subunit is a regulatory glycoprotein, which plays a role in the synthesis, stability, and the transport of α subunit of Na^+^/K^+^-ATPase [6].

The tissue-specific expression of Na^+^/K^+^-ATPase α and β subunits relate to cancer development. In human epithelial cancer cells, ATPase β1 isoform is frequently downregulated [9–13], while the α1-subunit level seems to have tissue-dependent change. For instance, the expression of ATP1A1 is significantly lower in prostate carcinoma than in normal and prostatic hyperplasia glands [9, 14], and its down-regulation of ATP1A1 leads to a decrease of the Na^+^, K^+^-ATPase activity in carcinoma [7]. However, in many non-small cell lung cancer samples, ATP1A1 was reported to have increased expression than in normal lung tissues [15]. In human hepatocellular carcinoma, ATP1A1 was known to promote carcinoma development [16]. Therefore, ATP1A1 expression varies in different tumor types.

The Na^+^/K^+^-ATPase α1 and β1 subunits are widely expressed in kidney [17, 18]. Na^+^/K^+^-ATPase β1 subunit is downregulated by its hypermethylated promoter [19], and it is also reported to suppress RCC tumor development by decreasing the phosphorelated ERK1/2 levels in Madin–Darby canine kidney cells [20]. However, ATP1A1 functions in RCC are not yet fully clear. In this study, we tend to investigate the ATP1A1 levels in patients’ RCC tissues using a quantitative proteomic approach. We also validate the data and reveal its biological roles and molecular mechanisms in RCC development.

## Methods

### Cell culture

HEK293 cells were cultured in DMEM medium (Corning, USA) with 10% dialyzed fetal bovine serum (FBS) (16000-044, Gibco, USA) through deuterated Leu (Leu-d_3_) based SILAC approach (stable isotope labeling with amino acids in cell culture) [21, 22]. The renal cancer cells OS-RC-2 and 786-0 were cultured in RPMI-1640 medium (Corning, USA) contained 10% FBS, and 100 U/ml penicillin. Cells were incubated in 37 °C with 5% CO_2_ and 95% air.

### Expression plasmid

ATP1A1 cDNA (GI: 237681107) and an expression vector pYR were double digested with restriction enzymes *Sgf*I and *Mlu*I separately, and then ligated with T4 ligase at 25 °C for 2 h. The ligated mixture was transformed into competent *E. coli* DH5α cells. The recombinant plasmid pYR-ATP1A1 was selected from LB agar with 50 µg/ml kanamycin, which was confirmed by DNA sequencing.

### Tissue samples

Eighty pairs of human clear cell renal cell carcinoma tissues (RCTs) and their autologous para-cancerous kidney tissues (PKTs) were obtained from West China Hospital, Sichuan University (Chengdu, P. R. China) with the offers’ informed consent guidelines established by the hospital. Prior review, consent, and approval for this project were provided by the Institutional Ethics Committee of State Key Laboratory of Biotherapy, West China Hospital of Sichuan University. All tissues were frozen in liquid nitrogen as soon as possible after surgical operation. The RCC patient’s clinical information, including the patient’s age, gender, and TNM stage [23], was collected with patient informed consent. The clinical information of 80 clear cell renal cell carcinoma (ccRCC) tissues was shown in detail in the Additional file 1: Table S1.

### Protein extraction and protein identification by MS

Total proteins from SILAC-labeling HEK293 cells and RCC, PKT tissues were prepared according to our previous reports [21, 22]. 30 µg cellular proteins from HEK293 cells were respectively mixed with equal proteins from RCTs and PKTs, and two group of protein mixture was isolated by SDS-PGAE. The 110-kDa band was cut to digest and peptides were identified by LC-nanospray-tandem mass spectrometry (MS/MS) using a QSTAR XL mass spectrometer (Applied Biosystems, USA). The relative protein expression level was quantified by tracking pairs of labeling and unlabeling peptides from the MS spectra.

### Cell proliferation

3 × 10^3^ OS-RC-2 or 786-0 cells were seeded in each well for a 96-well plate, then cells were transfected with 100 ng pYR-ATP1A1 plasmids or the empty vector pYR (Control) per well with Lipofectamine 2000 reagent (Invitrogen, Carlsbad, USA), and the mock group was only treated with the same volume of Lipofectamine 2000 reagent. After incubation for 24,48 72 and 96 h, 10% CCK-8 reagent (ZP328-3, Zomanio, China) was added to incubate for another 2 h at 37 °C. The optical density values (OD) were measured at 450 nm. Three independent experiments were performed. The data were calculated as mean ± SD. The comparisons among multiple groups were analyzed by Dunnet-t test. The statistical significance was defined as P < 0.05.

### Boyden chamber assay for cell migration

Cell migration was performed through Boyden chamber assay with 8 μm pore filters (PIEP12R48, Millipore, USA), which has been applied before [24, 25]. For cell migration assay, cells were cultured in a 6-well plate to transfect with 2.5 ug pYR-ATP1A1 or pYR plasmid with Lipo2000 reagent for 48 h. Then 500 μl serum-free medium was added into the upper chambers, and 500 μl medium containing 10% FBS was added into the lower chambers. 5 × 10^4^ transfected cells were added to the upper chambers to incubate for 24 h, and non-migrating cells were completely removed. Migratory cells were fixed by methanol, and stained with Giemsa (cat.# C0121, Beyotime, China). Cells were imaged and counted in five random fields under an Olympus inverted microscope (Lake Success, NY, USA) at 10× magnification. Cell experiment was performed in triplicate.

### Measurement of cell apoptosis and ROS production

After OS-RC-2 and 786-0 cells were transfected with pYR-ATP1A1 or pYR plasmids for 48 h, cells were harvested and washed with PBS buffer (8 g/l NaCl, 0.2 g/l KCl, 1.44 g/l Na_2_HPO4, 0.24 g/l K_2_HPO4, pH 7.4) supplemented with 1% bovine serum albumin (BSA) (P0010S-3, Beyotime, China). Cells were double stained with annexin V-FITC and propidium iodide (PI) (C1063, Beyotime, China) for apoptosis detection [26, 27], and production of ROS was measured by a single staining with DCFH-DA kit (S0033, Beyotime, China). Finally cells were observed by a flow cytometry (NovoCyte, ACEA Biosciences). Each experiment was performed in triplicate.

### Western blotting

OS-RC-2 and 786-0 cells were transfected with pYR-ATP1A1 or pYR plasmids for 48 h, and cell pellets were collected. Cell pellets and grated tissues were dissolved with RIPA buffer (Cat.# P0013C, Beyotime, China) to extract proteins. 100 μg proteins were separated on 10% SDS-PAGE and transferred onto polyvinylidene difluoride (PVDF) membrane (Cat.# IPVH00010, China). After blocking with 5% skim milk (OXOID), the PVDF membrane was incubated with primary antibodies overnight at 4 °C. The primary antibodies against ATP1A1 (1:200, ab2872, Abcam, UK), MEK1/2 (1:500, sc436, Santa Cruz, USA), p-MEK1/2(1:500, sc-81503, Santa Cruz, USA), ERK (1:500, sc-292838, Santa Cruz), p-ERK (1:500, sc-136521, Santa Cruz) β-actin (1:500, sc-1616, Santa Cruz, USA), and GAPDH (1:1000, sc-365062, Santa Cruz) were respectively diluted in TBST buffer (50 mM Tris–HCl, with 150 mM NaCl, 0.1% Tween-20, pH 7.4). Then the PVDF was incubated with the HRP-tagging secondary antibodies (ZSGB-BIO, China) at 37 °C for 1 h. Signals were finally detected with Western blot reagent ECL (Amersham Biosciences).

### Immunohistochemistry

Eighty pairs of RCC tissues (RCTs) and their counterparts, autologous para-cancerous kidney tissues (PKTs), were cut into 5-µm thickness sections for hematoxylineosin (HE) and immunohistochemistry (IHC) analysis according to our previous protocols [28]. The anti-ATP1A1 antibody (1:400, ab2872, Abcam, UK) was used to detect ATP1A1 level among RCTs and PKTs. A biotinylated anti-goat IgG (ZB-2306, ZSGB-BIO Corp., Beijing, China) was used as a secondary antibody. Then reaction with 3,3′-diaminobenzidine substrate solution (Dako Cytomation GmbH) and counterstaining with hematoxylin were performed. Five independent fields at ×50 magnification for positive cells were chosen to evaluate the intensity and percentage. The staining intensity was scored as 0 (negative), 1 (weak), 2 (moderate), or 3 (strong). The staining percentage was scored as 0 (negative), 1 (1–25%), 2 (26–50%), 3 (51–75%), and 4 (76–100%). The immunoreactivity score for each tissue sample, ranging from 0 to 12, was measured as immunostaining intensity multiplied by percentage of positive cells [24, 25]. The expression level of ATP1A1 was defined as our previous work [29]. The score of 0 was defined as negative, scores of 1–4 as low expression, and scores over 4 as high expression. The final score was an average value calculated from evaluation scores by two pathologists separately.

### Association of ATP1A1 expression with RCC clinical information

The association between ATP1A1 levels with clinic development of RCC was statistically analyzed by Pearson’s χ^2^ test mainly referencing our previous methods [25, 29]. The clinic information included patient’s gender, age, tumor stage, tumor size, and patient prognosis. According to the ATP1A1 expression level, 80 RCC patients were classified into two groups, ATP1A1-negative expression (Negative, n = 33) and ATP1A1-positive expression (Positive, n = 47). The patient overall survival was evaluated by Kaplan–Meier method. And a log-rank test was used to determine the statistical significance.

### Statistical analysis

The Pearson χ^2^ test was used to compare qualitative variables, and quantitative variables were analyzed by the Student’s t test when only two groups were compared. The comparisons among multiple groups were analyzed by Dunnet-t test. Data were calculated as mean ± SD. The statistical significance was defined as P< 0.05.

## Results

### ATP1A1 is downregulated in RCC

We used the spike-in SILAC approach [30] to compare the expression level of ATP1A1 between RCTs and PKTs [22]. Briefly, aliquots of RCTs and PKTs proteins were respectively mixed with equal amount of Leu-d_3_-labeled cell proteins, and were sent to SDS-PAGE separation. The 110-kD gel bands were processed for LC–MS/MS analysis following standard in-gel digestion protocol [22, 31]. The change of protein expression level between two tissue samples was determined by calculating the ratio of the two SILAC ratios, SILAC ratio 1/SILAC ratio 2. The SILAC ratio 1 was defined as the peak intensity of RCT proteins *versus* cell proteins (RCTs/HEK293), and the SILAC ratio 2 was defined the peak intensity of PKT proteins *versus* cell proteins (PKTs/HEK293).

**Fig. 1:**
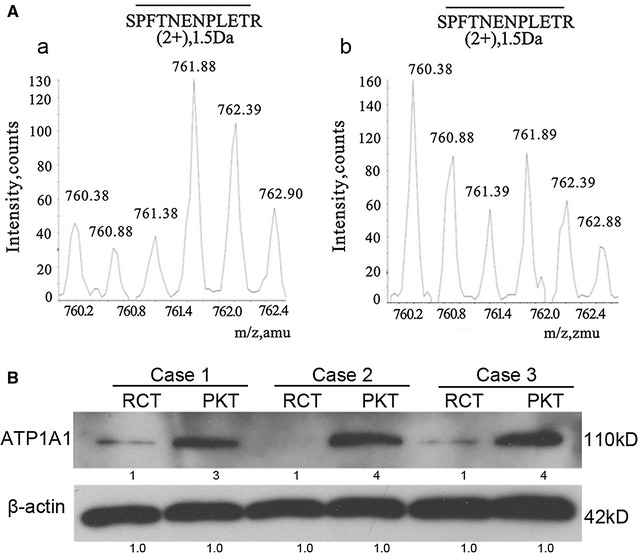
ATP1A1downregulation renal cell carcinoma. **A** One representative pair of isotope peptides of ATP1A1 with the sequence “SPFTENPLETR” (m/z 760.38/761.88, 2+) was used to quantify its differential expression level between human RCTs and PKTs. The mass spectra of this peptide from the protein mixture of PKTs and labeling HEK293 cells (*a*). The mass spectra of this peptide from sample mixture of RCTs and labeling HEK293 cells (*b*). The β-actin was taken as a loading control. **B** Analysis of relative expression of ATP1A1 in renal cell carcinoma tissues (RCTs) and autologous para-cancerous kidney tissues (PKTs) by Western blot

The protein ATP1A1 was successfully identified by our proteomic approach. One representative pair of isotope peptides in MS (m/z 760.38 versus m/z 761.88, 2+) was shown to quantify ATP1A1 between RCTs and PKTs (Fig. 1A; Table 1). The SILAC ratio 1 of ATP1A1 (RCTs/HEK293) was 0.45(Fig. 1a), and the SILAC ratio 2 (PKTs/HEK293) was 1.68 (Fig. 1A-b). Therefore, the change ratio of ATP1A1 expression (SILAC ratio 1/SILAC ratio 2) was 0.27 between RCTs and PKTs, which indicated that this protein was a 3.7-fold decrease in RCC tissues compared to noncancerous counterparts. While the value for the housekeeping protein β-actin was 1.06 between two states of tissues, which suggested its expression level was similar (Table 1).

**Table 1:** Quantitative identification of ATP1A1 expression in renal carcinoma

Protein	MOWSE scores (queries match)		No. of Leu-containing peptide^c^		SILAC-ratio^d^ (mean ± SD)		Change ratio^e^ (ratio 1/ratio 2)
	RCT^a^	PKT^b^	RCT^a^	PKT^b^	Ratio 1	Ratio 2	
ATP1A1	283 (15)	817 (38)	1	1	0.45	1.678	0.27
β-actin	380 (30)	288 (23)	5	5	1.01 ± 0.09	0.95 ± 0.08	1.06

^a^The protein mixture from RCT and Leu-d_3_ labeling HEK293 cells; *RCT* renal carcinoma tissue

^b^The protein mixture from PKT and Leu-d_3_ labeling HEK293 cells; *PKT* para-cancerous kidney tissues

^c^The number of unique Leu-containing isotope peptides used for MS quantification

^d^Average SILAC-ratio, from the intensity between tissues versus labeling HEK293 cells, was quantified using Analyst QS software. SILAC ratio 2: RCT/HEK293; SILAC ratio 1: PKT/HEK293

^e^The change ratio between two tissues (SILAC ratio 1/SILAC ratio 2)

We further validated the MS quantification on ATP1A1 by western blotting. ATP1A1 expression level was greatly decreased in three randomly chosen RCTs than corresponding PKTs (Fig. 1B).

### ATP1A1 expression is widely decreased in 80 RCC tissues

**Fig. 2:**
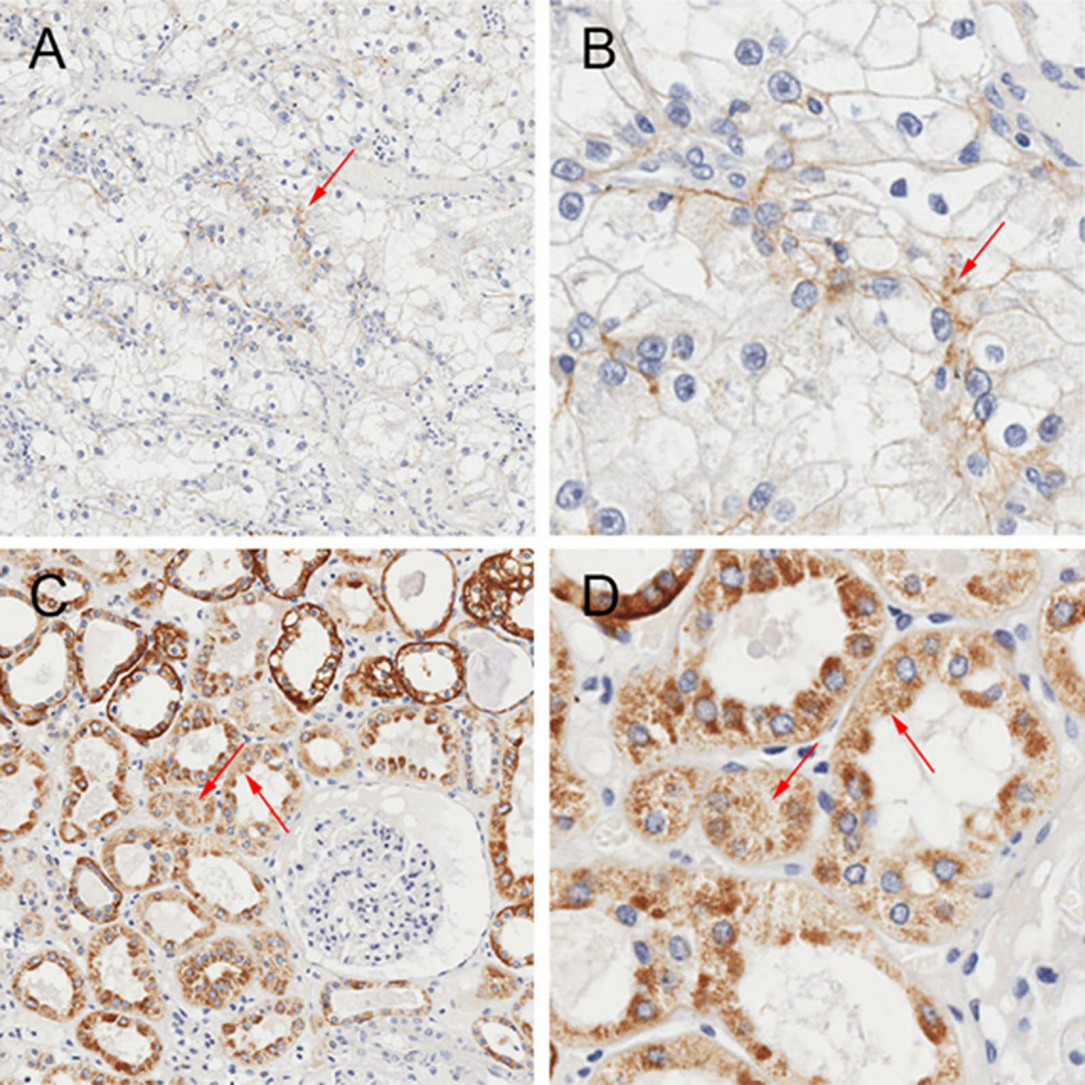
ATP1A1 expression in RCTs (**A**, **B**) and PKTs (**C**, **D**) detected by immunohistochemical analysis. **B**, **D** is respectively enlarged based on **A**, **B**. The *red arrow* means the positive staining cells. *RCTs* renal cell carcinoma tissues, *PKTs* autologous para-cancerous kidney tissues

The ATP1A1 expression profiling was analyzed in human RCC tissues from surgical resection by a tissue array, in which 80 pairs of RCTs and PKTs were detected by IHC analysis. In total, 33 RCTs (41.25%) have a negative ATP1A1 immuno-reactivity, 47 RCTs (58.75%) have a low ATP1A1 immunoreactivity with average scores 2.16 ± 1.98 (Table 2; Fig. 2A, B). On the other hand, 79 cases of PKTs (98.75%) showed a high level of ATP1A1 with average scores 8.46 ± 2.83 (Table 2; Fig. 2C, D), and only 1 case (1.25%) showed a negative staining. In PKTs, ATP1A1 was predominately expressed in cytoplasm of the convoluted tubules (Fig. 2C, D). Generally, 80 RCTs displayed a significantly lower expression of ATP1A1 with scores 1.27 ± 1.85 compared with the higher expression in PKTs with scores 8.35 ± 2.96 (P < 0.05) (Table 2).

**Table 2:** ATP1A1 immunoreactivity between RCTs and PKTs

Immuno-reactivity	RCTs (n = 80)			PKTs (n = 80)			P value
	Percentage	Total score	Average score	Percentage	Total score	Average score	
Total	100% (80/80)	101.5	1.27 ± 1.85	100% (80/80)	668	8.35 ± 2.96	
Negative	41.25% (33/80)	0	0 (−)	1.25% (1/80)	0	0 (−)	<0.05
Positive	58.75% (47/80)	101.5	2.16 ± 1.98 (+)	98.75% (79/80)	668	8.46 ± 2.83 (++)	

*RCTs* RCC tissues, *PKTs* para-cancerous kidney tissues

P value was calculated the differences between ATP1A1-positive RCTs and ATP1A1-negative PKTs using Student’ s t test. P < 0.05 was considered statistically significant

Negative: ATP1A1-negative expression with score 0; positive: ATP1A1-positive expression with score more than 0

−: Negative; +: low expression (the score was 1–4); ++: high expression (the score was over 4)

### ATP1A1 downregulation correlates with RCC malignant grade and patient’s poor survival

We studied the correlations between ATP1A1 expression and RCC clinicopathologic features. Among the 80 RCC patients, the expression of ATP1A1 had no obvious differences for gender, age and tumor size (P > 0.05). However, the decreased expression level of ATP1A1 in RCC is negatively associated with the tumor malignant degrees (TNM stage) with statistical significance. Totally 80 RCC patients included 10 patients with TNM III–IV and 70 ones with TNM I–II. Among 10 patients with TNM III–IV, 3 patients had a positive ATP1A1 expression while other 7 patients showed a negative expression. Meanwhile in 70 cases with TNM I–II, 44 of them showed a positive ATP1A1 level, and 26 of them showed a negative ATP1A1 expression. Therefore, the Pearson χ^2^ test indicated that ATP1A1 downregulation correlates with a higher RCC malignant grade (P < 0.05) (Table 3). A high percentage of loss or downregulation of ATP1A1 exists in RCC patients with TNM III–IV compared with the cases with TNM I–II.

**Table 3:** Correlations of ATP1A1 with RCC patient’s clinical information

Clinicopathologic variables	Case number	ATP1A1 protein		P value
		Negative (n = 33)	Positive (n = 47)	
Gender				
Male	44	22	22	0.079
Female	36	11	25	
Age				
<60	43	22	21	0.052
≥60	37	11	26	
Tumor stage				
I–II	70	26	44	0.038
III–IV	10	7	3	
Tumor size				
<100	46	17	29	0.364
≥100	34	16	18	

P < 0.05 was considered statistically significant. P value was calculated using Pearson χ^2^ test

Negative: ATP1A1-negative expression with score 0; positive: ATP1A1-positive expression with score more than 0

**Fig. 3:**
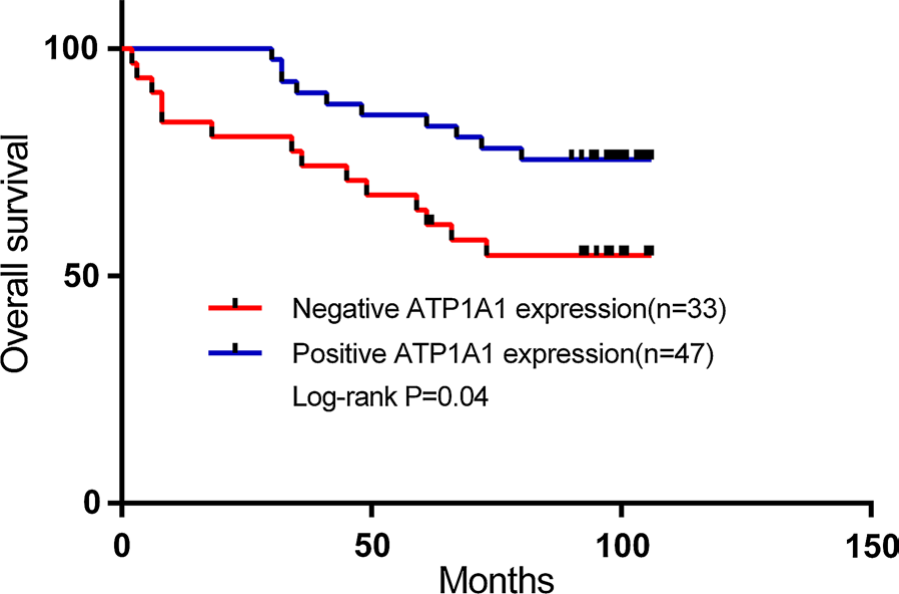
Associations between ATP1A1 expression and the overall survival period of RCC patients. Kaplan–Meier survival analysis showed that patients with negative ATP1A1 expression have a shorter overall survival than ones with positive ATP1A1 level (P < 0.05)

Furthermore, we investigated the relationship between ATP1A1 expression and RCC patient’s survival time. The Kaplan–Meier analysis indicated RCC patients with a positive ATP1A1 expression had a significant longer survival time than those patients with a negative expression (P = 0.04 by the log-rank test, Fig. 3). The average survival among patients with ATP1A1-negative expression was 69 ± 36 months, while among patients with ATP1A1-positive expression, the average survival was 87 ± 22 months. Generally, the decreased expression level of ATP1A1 in RCC is associated with a poor prognosis of patients.

### Overexpression of ATP1A1 inhibits RCC cell proliferation and cell migration

**Fig. 4:**
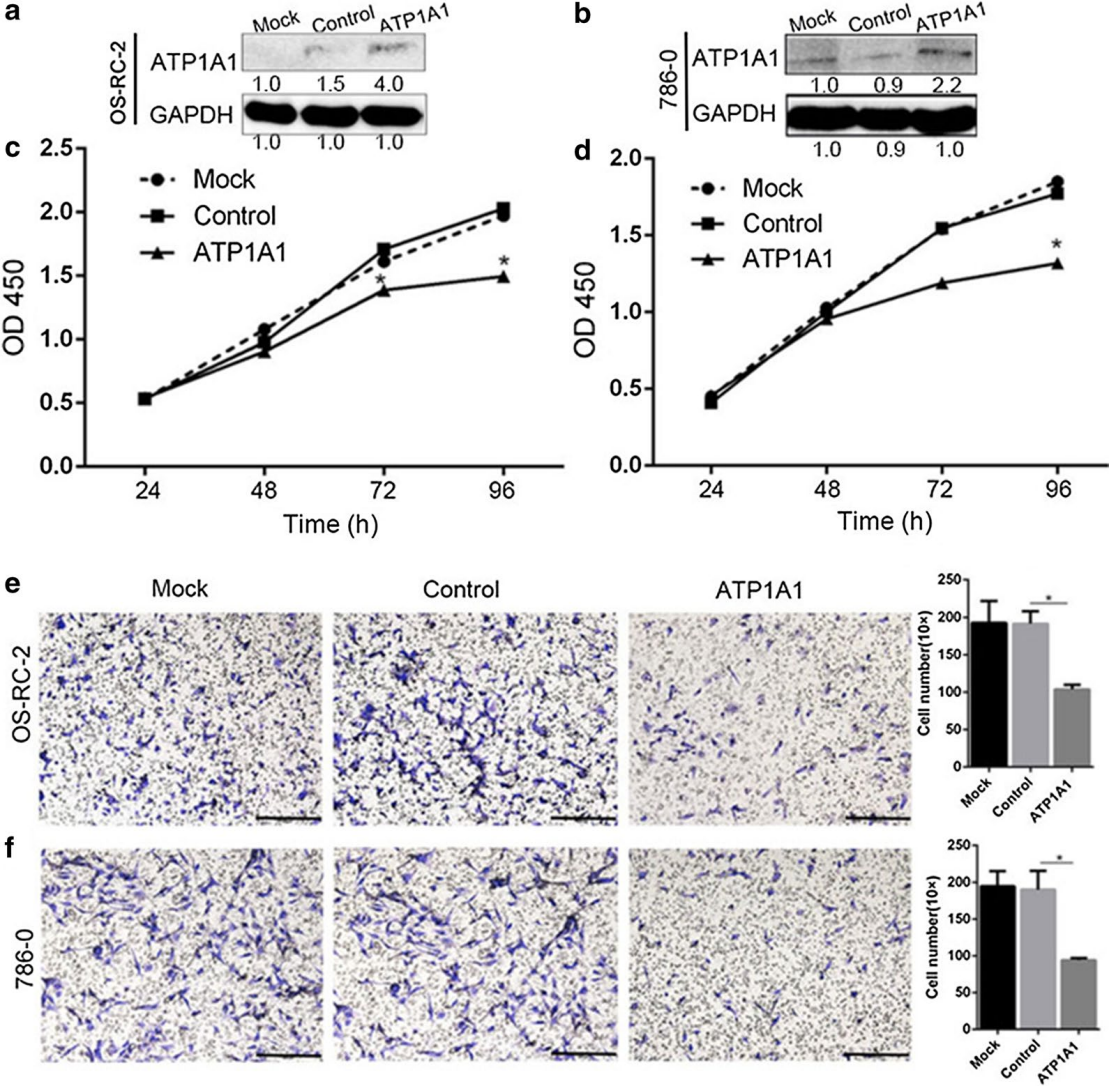
ATP1A1 overexpression inhibits cell proliferation and cell migration of RCC cells. **a**, **b** Expression level of ATP1A1 was determined by Western blot in RCC cells transfected with pYR-ATP1A1 plasmids (ATP1A1) or the empty pYR vector (Control). Mock: RCC cells without transfection. **c**, **d** Cell proliferation was detected at transfection for 0, 24, 48, 72 h in RCC cells. The experiment was performed in triplicate. *P < 0.05. **e**, **f** Cell migration of OS-RC-2 and 786-0 cells transfected with pYR-ATP1A1 plasmids (ATP1A1) or the empty vector (Control) by a transwell assay. *Scale bar* 100 μm (original magnification ×200). The experiment was performed in triplicate, and data were shown as mean ± SD, *P < 0.05

We performed gain-of-function experiments of ATP1A1 to investigate this protein influence on RCC cell growth and migration. The endogenous ATP1A1 expression was low in OS-RC-2 and 786-0 cells (Fig. 4a, b). When cells were transiently transfected with pYR-ATP1A1 plasmids for 72–96 h, cell proliferation was markedly lower than cells transfected with the empty pYR vector (P < 0.05) (Fig. 4c, d). The data indicated that ATP1A1 inhibits cell proliferation of RCC cells.

Correspondingly, the migrating cell number was about 99, 92 per well for OS-RC-2 and 786-0 cells transfected with pYR-ATP1A1 plasmids for 48 h, while cell number was proximate 218, 209 per well in the mock group and 203, 208 per well in the control group in which cells transfected with the empty pYR plasmids. The number of migrating cells was significantly decreased by 50% due to the exogenous overexpression of ATP1A1 in OS-RC-2 (Fig. 4e) and 786-0 cells (P < 0.05) (Fig. 4f).

### Overexpression of ATP1A1 promotes ROS production and cell apoptosis

**Fig. 5:**
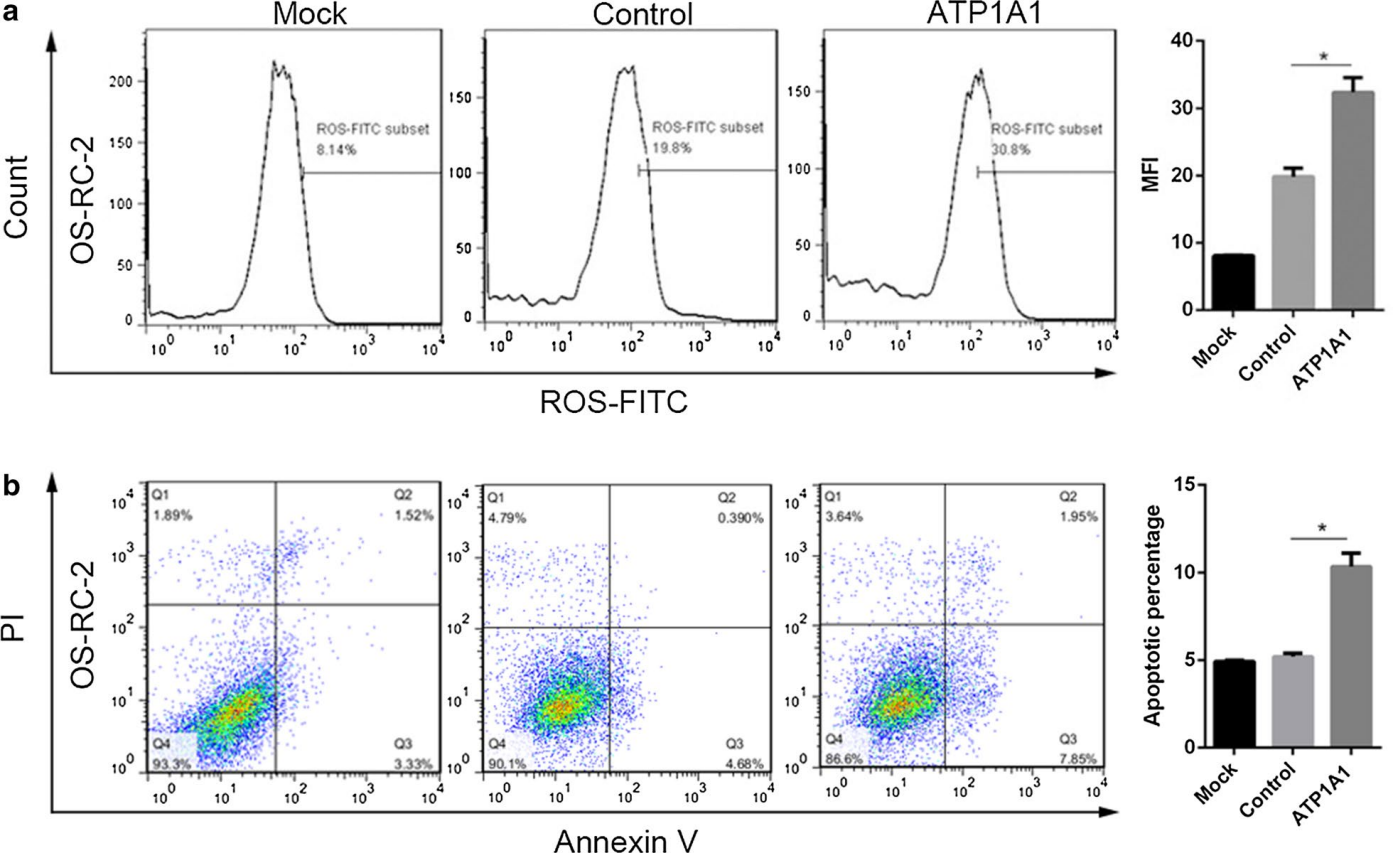
ATP1A1 overexpression induces the ROS generation (**a**) and cell apoptosis (**b**) in OS-RC-2 cells. Cells were harvested after transfection with pYR-ATP1A1 plasmids (ATP1A1) or the empty pYR vector (Control) in RCC cells for 48 h. **a** ROS generation was increased in OS-RC-2 cells. The experiment was performed in triplicate, *P < 0.01. **b** Cell apoptosis was detected by Annexin V-PI double staining and flow cytometry analysis. The experiment was performed in triplicate, *P < 0.05

To further explore how the overexpression of ATP1A1 inhibits cell growth for RCC cells, we measured cell apoptosis and ROS production. We found, the ROS production was increased to 30.9% in OS-RC-2 cells transiently transfected with pYR-ATP1A1 plasmids for 48 h, compared with the mock cells (8.14%) and the control cells treated with empty vector (19.8%) (Fig. 5a). And under same conditions, the ROS production was also elevated from 41.8 to 52.9% in ATP1A1-overexpressing 786-0 cells (data not shown).

Previous work illustrated that the accumulation of ROS leads to DNA damage [32], which triggers cell growth inhibition and cell death to prevent cancer development in early tumorigenesis [33, 34]. Similarly we found that cell apoptosis was increased in ATP1A1-overexpressing cells. When ROS appeared after transfection with pYR-ATP1A1 plasmids for 48 h in OS-RC-2 cells, cell apoptosis was 10.34%, which was obviously increased when compared with 4.91 and 5.20% cell apoptosis in mock and control group (Fig. 5b). The same result was obtained in 786-0 (data not shown). These results indicated that ATP1A1 overexpression induced cell apoptosis possibly due to ROS production in RCC cells, which finally inhibited cell growth.

### ATP1A1-mediated Raf/MEK/ERK signaling pathways in RCC cells

**Fig. 6:**
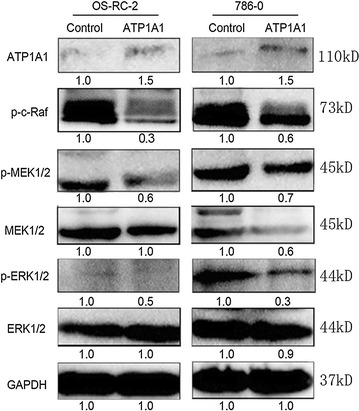
ATP1A1-mediated Raf/MEK/ERK signaling pathways in RCC cells. RCC cells were collected at 48 h after transfection with ATP1A1-expressing plasmids pATP1A1 or the control empty vector (Control). The phosporylation level of Raf/MEK/ERK was decreased along with ATP1A1 upregulation in RCC cells. The experiment was performed in triplicate

To understand ATP1A1-mediated molecular signaling in RCC cells, we detected the protein levels in ATP1A1-involved Raf/MEK/ERK pathway in RCC cells. We found that the expression levels of the phosphorylated MEK1/2 and ERK (p-MEK1/2, p-ERK) were significantly decreased to 60 ± 0.05, 50 ± 0.08% in ATP1A1-overexpressing OS-RC-2 cells, or decreased to 70 ± 0.08 and 30 ± 0.04% in ATP1A1-overexpressing 786-0 cells (P < 0.05) (Fig. 6). The upstream protein, the phosphorylated Raf, was also respectively downregulated to 30 and 70% in MEK/ERK signaling cascade in OS-RC-2 and 786-0 cells.

## Discussion

As we know, Leucine is one of the most common amino acids found in protein sequences. Leu residue constitutes about 9.1% in human proteome, and the average length of a tryptic peptide is around 11 amino acids [35]. Therefore, in theory each tryptic peptide may contain one Leu. In addition, comparing with Arg/Lys-based labeling approach, Leu-d_3_ is much more cost-effective (around 8–10 times cheaper than Arg6/Lys8, according to Sigma-Aldrich prices), appealing to many laboratories that have limited financial support. This is one of the reasons we chose heavy Leu as a labeling reagent. Regarding the Leu/Ile issue, as the reviewer is already aware of, some proteomic software couldn’t distinguish these two amino acids and treat them identically when doing database search. However, the Leu-d3 based labeling can efficiently differentiate them. Whenever there is a Leu in a tryptic peptide, its labeled counterpart should be present adjacently on mass spectrum, whereas the isoleucine doesn’t have such pairwise peak. The advantage of using labeled Leu to assist De Novo peptide sequencing and to differentiate Leu/Ile has been discussed before [35]. Traditional Arg/Lys based SILAC is certainly advantageous owing to the fact that each tryptic peptide is ended with either one of them. In the case of global or large-scale proteomic quantitation, this makes the mass spectrum data fully utilized for SILAC quantitation. In our case, only a couple of targeted proteins were quantified. The current Leu based SILAC served well for our purpose.

ATP1A1 expression is usually incresased in some types of cancers, such as lung cancer, liver cancer, glioblastoma and melanoma [15, 16, 36, 37]. However, in our study, we confirmed that the expression level of ATP1A1 is generally lower in ccRCC tissues than in adjacent nontumor counterparts based on a quantitative proteomics identification and integrative biological assessments. This result is consistent with previous work [38, 39]. For example, ATP1A1 mRNA is reported to decrease in RCC [38]. And Seligson DB’s work also confirmed that ATP1A1 level is decreased in ccRCC tissues [39]. In their study, the intermediate expression of ATP1A1 was scored from 2 to 2.4, and the low expression was scored less than 2. It is interesting the estimated median disease free survival for ATP1A1 intermediate, low and high expressers was 10.4, 4.8 and 1.7 years respectively [39], which demonstrates RCC patients with an intermediate ATP1A1 expression level have much longer overall survival and better prognosis than those patients with a low or high ATP1A1 expression level. Actually the conclusion is consistent with our data on the associationship of ATP1A1 downregulation and RCC overall survival. By now in our limited objects, only 47 RCC cases have ATP1A1-positive expression with average scores 2.16, which just accords with the scoring group of the intermediate ATP1A1 expression in Seligson DB’s report [39]. While the other 33 cases have ATP1A1-negative level (Table 2). So far the RCC patients with ATP1A1-positive expression have a longer overall survival than the ATP1A1-negative expressers. In our future study, more RCC patients with TNM III–IV will be recruited to further investigate the protein linkage with RCC grade subcategory.

Although the ATP1A1 is greatly decreased even lost in RCC tissues, it is accurate to catch its downregulation by the sensitive quantitative proteomics identification, which provides a rapid approach to discover novel cancer biomarkers [40]. Our data on ATP1A1 expression is consistent with previous work [38, 39]. Similarly, ATP1A1 level is also decreased in colorectal cancer [7]. More importantly, our research demonstrates the loss or downregulation of ATP1A1 relates with higher RCC tumor grades and patient’s poor prognosis. RCC patients with ATP1A1-positive level exhibit a longer overall survival time than the ATP1A1-negative patients. It has been attempted to combat glioblastoma cells by targeting the ATP1A1 in an orthotopic human glioblastoma xenograft model [36]. Similarly, targeting ATP1A1 is a novel approach to the treatment of HCC [16]. So far, ATP1A1 is a promising tumor biomarker to develop antitumor drug or to monitor therapy efficiency.

**Fig. 7:**
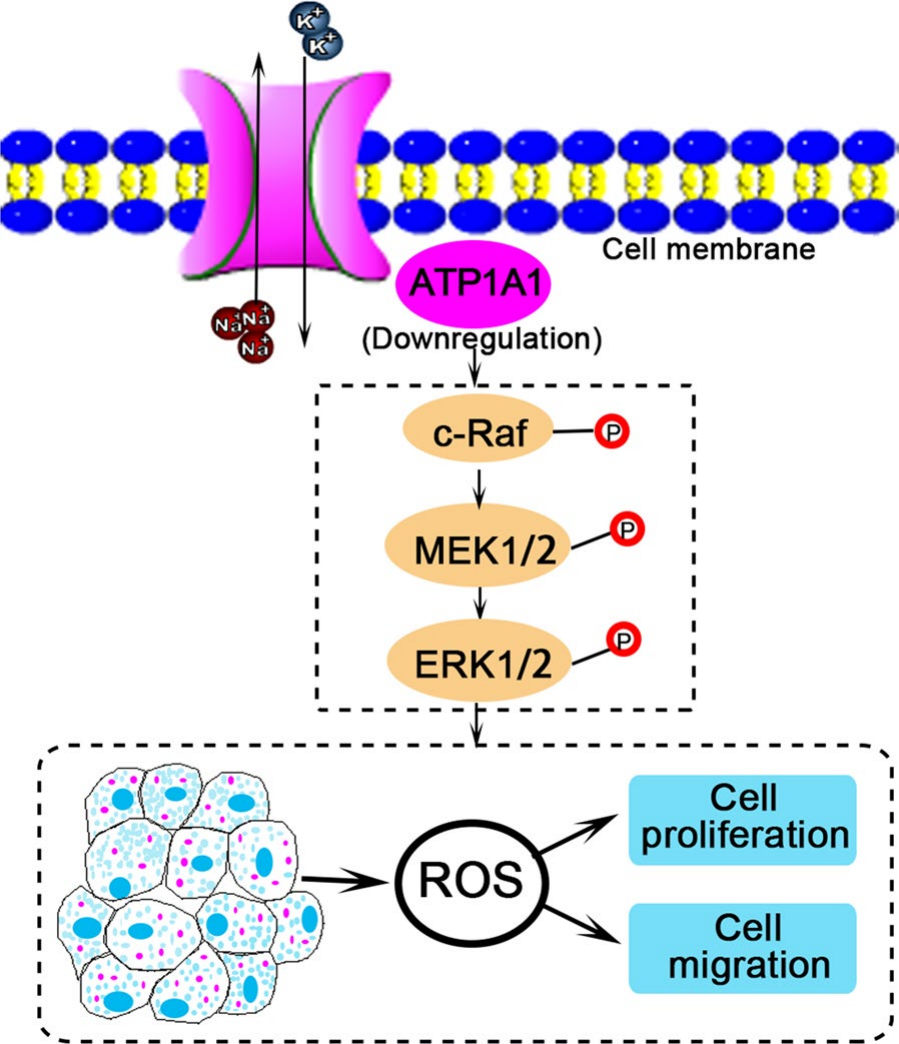
Schematic summary of ATP1A1 roles in RCC development. The endogenous downregulation of ATP1A1 in RCC cells promotes the phosphorylation of c-Raf, MEK1/2 and ERK1/2, which affects ROS generation and increases cell growth and cell migration. *RCC* renal cell carcinoma. The  means phosphorylation

ATP1A1 is a multifunctional protein that displays different roles in cell junctions, adhesion, motility and signal transduction [16, 41]. We have confirmed that the exogenous upregulation of ATP1A1 inhibits RCC cell proliferation and cell migration possibly by increasing ROS production, and finally induces cell apoptosis, in which ATP1A1-mediated Raf/MEK/ERK signaling pathway is suppressed in RCC cells (Fig. 7). We can assume that the endogenous decrease of ATP1A1 in RCC induces cell growth and migration by activating Raf/MEK/ERK signaling, while its upregulation by exogenous overexpressing inhibits the phosphorylation of Raf/MEK/ERK to induce cell apoptosis. These findings are the direct evidences to show ATP1A1 inhibition roles as a tumor suppressor in RCC. High levels of ROS have been found to promote the mtDNA damage and subsequent mitochondrial dysfunction in senescence cells [42]. It is also proved that oxidative stress can lose cell proliferation in human fibroblasts [43]. Although ATP1A1 gene expression is markedly higher in HCC samples than in adjacent nontumor tissue samples, the abnormal ATP1A1 expression is validated to cause intracellular accumulation of ROS in HCC cells [16].

As MEK/ERK pathway plays significant roles in cell growth [44], we have explored ATP1A1-mediated Raf/MEK/ERK pathway to regulate cell growth and apoptosis in RCC cells. Due to the binding site for ATP and phosphorylation site on the cytoplasmic domain, ATP1A1 is considered as a catalytic subunit [41]. By now, it is not clear how ATP1A1 affects phosphorylation of Raf/MEK/ERK in RCC cells. The ATP1A1-mediated molecular mechanism still needs to be clarified in detail.

## Conclusions

In summary, we have successfully detected the decreased level of ATP1A1 in RCC tissues by a sensitive proteomic approach and integrative biochemical assessment. RCC patients with ATP1A1-positive expression have a longer overall survival time than the ATP1A1-negative patients. Furthermore, the exogenous expression of ATP1A1 inhibits RCC cell proliferation and cell migration possibly by increasing ROS production, and induces cell apoptosis, in which the phosphorylation of Raf/MEK/ERK is obviously suppressed in RCC cells (Fig. 7). Our work indicates ATP1A1 is a novel potential suppressor protein for renal cancer based on ATP1A1 inhibition roles for RCC progression in vitro and in vivo.

## Additional file

**Additional file 1: Table S1.** The detailed information from 80 pairs of RCC tissues in this work..

## Abbreviations

ATP1A1: Na^+^/K^+^-ATPase α1 subunit
HCC: hepatocellular carcinoma
IHC: immunohistochemistry
Leu-d_3_: deuterated Leu
PKTs: autologous para-cancerous kidney tissues
RCC: renal cell carcinoma
RCTs: RCC tissues
ROS: reactive oxygen species
SILAC: stable isotope labeling with amino acids in cell culture
