# Supplementary material for: Downregulation of ATP1A1 promotes cancer development in renal cell carcinoma
Source: Clin Proteomics. 2017 May 4;14:15. doi: 10.1186/s12014-017-9150-4 (PMC5418755; doi:10.1186/s12014-017-9150-4)
Supplement: Supplementary file 1 — Additional file 1: Table S1. The detailed information from 80 pairs of RCC tissues in this work.. [file 12014_2017_9150_MOESM1_ESM.docx]

Table S1. Detailed information on ccRCC tissues

| Number | Types of tumor | Treating | Tumor location | T Category Changes | Nodes (N) | Metastases (M) | Tumor stage |
| --- | --- | --- | --- | --- | --- | --- | --- |
| 1 | ccRCC | RN | RK | T2a | N0 | M0 | 2 |
| 2 | ccRCC | RN | LK | T1b | N0 | M0 | 1 |
| 3 | ccRCC | RN | LK | T1b | N0 | M0 | 2 |
| 4 | ccRCC | RN | RK | T1a | N0 | M0 | 1 |
| 5 | ccRCC | RN | LK | T2a | N0 | M0 | 3 |
| 6 | ccRCC | RN | LK | T1a | N0 | M0 | 2 |
| 7 | ccRCC | RN | LK | T2a | N0 | M0 | 2 |
| 8 | ccRCC | RN | RK | T2a | N0 | M0 | 2 |
| 9 | ccRCC | RN | LK | T1b | —— | M0 | 1 |
| 10 | ccRCC | RN | RK | T2a | N0 | M0 | 2 |
| 11 | ccRCC | RN | LK | —— | N0 | M0 | 1 |
| 12 | ccRCC | RN | LK | T1b | N0 | M0 | 2 |
| 13 | ccRCC | RN | RK | T1b | N0 | M0 | 2 |
| 14 | ccRCC | RN | LK | T1a | N0 | M0 | 1 |
| 15 | ccRCC | RN | LK | T2a | N0 | M0 | 3 |
| 16 | ccRCC | RN | LK | T1a | N0 | M0 | 1 |
| 17 | ccRCC | RN | LK | T1a | N0 | M0 | 1 |
| 18 | ccRCC | RN | RK | T1a | N0 | M0 | 2 |
| 19 | ccRCC | RN | LK | T1a | N0 | M0 | 2 |
| 20 | ccRCC | RN | LK | T1b | N0 | M0 | 2 |
| 21 | ccRCC | RN | LK | T1b | N0 | M0 | 2 |
| 22 | ccRCC | RN | LK | T1a | N0 | M0 | 1 |
| 23 | ccRCC | RN | RK | T1b | N0 | M0 | 1 |
| 24 | ccRCC | RN | RK | T1a | N0 | M0 | 1 |
| 25 | ccRCC | RN | RK | —— | N0 | M0 | 2 |
| 26 | ccRCC | RN | LK | T1a | N1 | M0 | 3 |
| 27 | ccRCC | RN | LK | T2a | N0 | M0 | 2 |
| 28 | ccRCC | RN | LK | T2b | N0 | M0 | 3 |
| 29 | ccRCC | RN | RK | T2a | N0 | M0 | 2 |
| 30 | ccRCC | RN | LK | T1b | —— | M0 | 3 |
| 31 | ccRCC | RN | LK | T1a | N0 | M0 | 1 |
| 32 | ccRCC | RN | LK | T1a | N0 | M0 | 2 |
| 33 | ccRCC | RN | LK | T1a | N0 | M0 | 1 |
| 34 | ccRCC | RN | RK | —— | N0 | M0 | 2 |
| 35 | ccRCC | RN | LK | T1b | N0 | M0 | 2 |
| 36 | ccRCC | RN | LK | T1a | N0 | M0 | 1 |
| 37 | ccRCC | RN | RK | T1b | N0 | M0 | 1 |
| 38 | ccRCC | RN | RK | T1a | N0 | M0 | 2 |
| 39 | ccRCC | RN | RK | T1a | N0 | M0 | 1 |
| 40 | ccRCC | RN | RK | T3 | N0 | M0 | 3 |
| 41 | ccRCC | RN | LK | T1b | N0 | M0 | 1 |
| 42 | ccRCC | RN | RK | T1b | N0 | M0 | 2 |
| 43 | ccRCC | RN | RK | T2a | N0 | M0 | 2 |
| 44 | ccRCC | RN | LK | T1a | N0 | M0 | 2 |
| 45 | ccRCC | RN | RK | T2a | N0 | M0 | 2 |
| 46 | ccRCC | RN | RK | T3 | N0 | M1 | 3 |
| 47 | ccRCC | RN | RK | T2a | N0 | M0 | 2 |
| 48 | ccRCC | RN | RK | T1a | N0 | M0 | 1 |
| 49 | ccRCC | RN | LK | T3 | N0 | M0 | 1 |
| 50 | ccRCC | RN | RK | T1b | N0 | M0 | 4 |
| 51 | ccRCC | RN | LK | T1a | N0 | M0 | 1 |
| 52 | ccRCC | RN | RK | T1b | N0 | M0 | 2 |
| 53 | ccRCC | RN | LK | T1a | N0 | M0 | 2 |
| 54 | ccRCC | RN | RK | T1a | N0 | M0 | 2 |
| 55 | ccRCC | RN | RK | T1b | N0 | M0 | 2 |
| 56 | ccRCC | RN | RK | T1b | N0 | M0 | 2 |
| 57 | ccRCC | RN | RK | T1a | N0 | M0 | 2 |
| 58 | ccRCC | RN | RK | T3 | N0 | M1 | 2 |
| 59 | ccRCC | RN | LK | T1b | N0 | M0 | 1 |
| 60 | ccRCC | RN | RK | T1b | N0 | M0 | 2 |
| 61 | ccRCC | RN | RK | T2a | N0 | M0 | 3 |
| 62 | ccRCC | RN | LK | T1a | N0 | M0 | 1 |
| 63 | ccRCC | RN | RK | T2a | N0 | M0 | 3 |
| 64 | ccRCC | RN | RK | T1a | N0 | M0 | 2 |
| 65 | ccRCC | RN | RK | T1b | N0 | M0 | 1 |
| 66 | ccRCC | RN | RK | T1b | N0 | M0 | 1 |
| 67 | ccRCC | RN | RK | T1b | N0 | M0 | 2 |
| 68 | ccRCC | RN | RK | T1b | N0 | M0 | 1 |
| 69 | ccRCC | RN | RK | T1a | N0 | M0 | 1 |
| 70 | ccRCC | RN | RK | T1a | N0 | M0 | 2 |
| 71 | ccRCC | RN | LK | T1a | N0 | M0 | 2 |
| 72 | ccRCC | RN | LK | T1a | N0 | M0 | 1 |
| 73 | ccRCC | RN | RK | T1a | N0 | M0 | 1 |
| 74 | ccRCC | RN | RK | T2b | N0 | M0 | 2 |
| 75 | ccRCC | RN | RK | T1b | N0 | M0 | 1 |
| 76 | ccRCC | RN | LK | T1b | N0 | M0 | 2 |
| 77 | ccRCC | RN | LK | T1b | N0 | M0 | 1 |
| 78 | ccRCC | RN | RK | T1b | N0 | M0 | 2 |
| 79 | ccRCC | RN | LK | T1a | N0 | M0 | 1 |
| 80 | ccRCC | RN | LK | T1b | N0 | M0 | 2 |

ccRCC: clear cell renal cell carcinoma;

RN: radical nephrectomy;

LK: left kidney;

RK: right kidney;

T1: tumors invading subepithelial connective tissue;

T1a: tumors less than or equal to 4 cm;

T1b: tumors greater than 4 cm but less than or equal to 7 cm;

T2a: tumors greater than 7 cm but less than or equal to 10 cm;

T2b: tumors greater than 10 cm;

T3: renal vein involvemen and adjacent tissues;

N0: nodal involvement without invasion;

N1: single positive node in primary drainage regions;

M0: ipsilateral and adrenal involvement without distant invasion;

M1: ipsilateral and adrenal involvement with distant invasion.
